# Mass Azithromycin Distribution and Cause-Specific Mortality among Children Ages 1–59 Months Old: A Secondary Analysis of a Cluster-Randomized Controlled Trial

**DOI:** 10.4269/ajtmh.25-0482

**Published:** 2025-11-18

**Authors:** Ali Sié, Mamadou Ouattara, Mamadou Bountogo, Boubacar Coulibaly, Valentin Boudo, Thierry Ouedraogo, Elisabeth Gebreegziabher, Huiyu Hu, Elodie Lebas, Benjamin F. Arnold, Thomas M. Lietman, Catherine E. Oldenburg

**Affiliations:** ^1^ Centre de Recherche en Santé de Nouna, Nouna, Burkina Faso;; ^2^ Francis I. Proctor Foundation, University of California, San Francisco, California;; ^3^ Department of Epidemiology & Biostatistics, University of California, San Francisco, California;; ^4^ Department of Ophthalmology, University of California, San Francisco, California

## Abstract

Mass azithromycin distribution has been shown to reduce all-cause child mortality in several settings in the Sahel by 14–18%. A trial in Niger found that mass azithromycin distribution to children ages 1–59 months old reduced cause-specific mortality because of malaria, dysentery, meningitis, and pneumonia. However, this study was done in the absence of seasonal malaria chemoprevention (SMC). Here, we assess the effect of mass azithromycin distribution on cause-specific child mortality in a setting receiving SMC. The Child Health with Azithromycin Treatment trial was a cluster-randomized, placebo-controlled trial of 341 communities in Nouna District, Burkina Faso. Eligible children (ages 1–59 months old) received a single oral 20-mg/kg dose of azithromycin or matching placebo. Six rounds of distribution occurred over a 36-month period. An enumerative census was conducted during each twice-yearly distribution, during which vital status for all children in the community was collected. Verbal autopsy was performed to assess cause of death. Of 1,086 deaths recorded in the trial, verbal autopsy results were available for 992 (91%). The most common causes of death were infectious, including malaria (34%), diarrhea (24%), and pneumonia (9%). Children living in communities receiving azithromycin had significant reduction in malaria mortality (incidence rate ratio, 0.67; 95% CI, 0.50–0.90; *P* = 0.008). Other infectious causes of mortality, including diarrhea and pneumonia, were lower in communities receiving azithromycin but were not statistically significantly different. Mass azithromycin distribution for child mortality has benefits in the context of SMC for reducing mortality, including for malaria mortality.

## INTRODUCTION

Biannual mass azithromycin distribution has been shown in several cluster-randomized trials to reduce all-cause child mortality when distributed periodically throughout the year.^1^^,^^2^ In the Niger site of the Macrolides Oraux pour Reduire le Deces avec un Oeil sur la Resistance (MORDOR) study, a significant reduction in mortality owing to malaria, dysentery, meningitis, and pneumonia was observed in azithromycin communities compared with placebo.^3^ The MORDOR study was done in the absence of seasonal malaria chemoprevention (SMC), a strategy that involves monthly distribution of sulfadoxine-pyramethamine (SP) and amodiaquine (AQ) to children ages 3–59 months old during the high-malaria-transmission season in regions with highly seasonal malaria transmission.^4^ Seasonal malaria chemoprevention is highly effective at reducing the incidence of clinical malaria; however, the impact of SMC on mortality is less clear, with randomized, controlled trials generally showing no effect of SMC on all-cause mortality.^5^^,^^6^ After the results of MORDOR-Niger, debate ensued regarding whether mass azithromycin distribution would continue to be effective in other regions of the Sahel where SMC is routinely distributed as malaria is one of the most common causes of mortality in the region. Azithromycin has modest antimalarial properties through targeting the plasmodial apicoplast.^7^^,^^8^ In addition to results suggesting that azithromycin reduced malaria mortality in MORDOR, some studies have suggested that mass azithromycin distribution may reduce malaria parasitemia in Niger, including in MORDOR.^9^^,^^10^ Although at the individual level, azithromycin is not an effective antimalarial,^11^^,^^12^ mass distribution may lead to lower overall community transmission.

The Child Health with Azithromycin Trial (CHAT) was a cluster-randomized trial in Burkina Faso that evaluated mass azithromycin distribution in the presence of SMC.^13^ The trial was designed to be a replication of the MORDOR trial in a setting with lower anticipated mortality where SMC was being distributed. The CHAT found an 18% reduction in all-cause mortality in children ages 1–59 months old, a point estimate that was identical to that observed at the Niger site of MORDOR.^1^^,^^2^ Despite the similarity in the reduction in all-cause mortality, the presence of SMC may change the distribution of cause of death, and azithromycin distribution may have different effects on cause-specific mortality in the presence of SMC compared with the absence of SMC, particularly for malaria. Here, we evaluated whether biannual mass azithromycin distribution reduced cause-specific mortality as assessed by verbal autopsy in the CHAT trial.

## MATERIALS AND METHODS

### Study overview.

Complete methods for the CHAT trial have been previously published.^2^^,^^13^ In brief, CHAT was a cluster-randomized community trial in which 341 clusters in Nouna District, Burkina Faso were randomized to six rounds of twice-yearly mass distribution of azithromycin or matching placebo to children 1–59 months of age from August 2019 until February 2023. Clusters consisted of villages with <2,000 inhabitants or villages split into units with <2,000 inhabitants each for larger communities. The primary outcome for the trial was all-cause mortality, and it has been previously reported.^2^ Three hundred and forty-one clusters were randomized to azithromycin or placebo; however, some clusters were lost over the course of the study because of security concerns in the study area. This was anticipated and accounted for in the trial’s statistical analysis plan. In total, 285 clusters contributed person-time to the analysis. The study was reviewed and approved by the Institutional Review Board at the University of California, San Francisco; the Comité d’Ethique pour la Recherche en Santé in Ouagadougou, Burkina Faso; and the Comité Technique d’Examen des Demandes d’Autorisation d’Essais Cliniques, Ouagadougou. Written informed consent was obtained from at least one guardian of each included child.

### Study setting.

The CHAT was conducted in Nouna District, Burkina Faso. Nouna is in northwestern Burkina Faso near the border with Mali in the Sahel. The region experiences seasonal rainfall from approximately July through October, which coincides with the high-malaria-transmission season. Seasonal malaria chemoprevention with SP-AQ has been distributed monthly in July, August, September, and October in Nouna since 2014 through door-to-door distribution to children ages 3–59 months old. Seasonal malaria chemoprevention distribution, therefore, overlapped with azithromycin distribution from July to October in all years of the CHAT trial.

### Participants.

Children were eligible for treatment as part of the trial if they weighed at least 3,800 g at the time of the census and were between the ages of 1 and 59 months old. Children were followed up to 65 months of age (6 months after the last eligible treatment age) for vital status assessment.

### Randomization, interventions, and masking.

Communities were randomized before the first census and treatment round to either biannual mass azithromycin distribution or placebo. Randomization was stratified by whether the community was within an existing Health and Demographic Surveillance Site.^14^ Eligible children receive a single oral 20-mg/kg dose of azithromycin or equivalent volume of matching placebo (donated by Pfizer, Inc., New York, NY). All doses of study medication were directly observed. Study staff, investigators, participants, and caregivers were masked to the community’s randomized treatment allocation.

### Census.

Every 6 months, a door-to-door enumerative census was performed in each study community. During the census, the head of each household was interviewed to determine the number of children younger than 5 years of age residing in the household and the vital status of each child. Each census worker had a list of children in residence recorded during previous census rounds; workers updated the vital status (alive, died, moved, or unknown) for each known child and added any new children who joined the household since the last census round. Study treatment (described below) was provided to all children ages 1–59 months old at the time of the census.

### Cause of death assessment.

A list of children who died per the census was generated during each census phase. After a mourning period of approximately 3 months, a verbal autopsy interview was attempted with the caregiver or head of household for each child who died using the 2016 WHO Verbal Autopsy instrument for children ages 4 weeks to 11 years old. Verbal autopsy results was assigned by inSilicoVA, an algorithm that assigns most likely cause of death, using R statistical software (R Foundation for Statistical Computing, Vienna, Austria).^15^ The primary cause of death as assigned by the algorithm was used as the cause of death in all analyses.

## STATISTICAL ANALYSES

The sample size for the study was based on the primary outcome: all-cause mortality among children 1–59 months of age. Because the present analysis considered cause-specific deaths, we anticipated lower statistical power relative to the primary outcome, and we anticipated that we would only be able to detect relatively large effect sizes. The overall distribution of causes of death was compared between arms using multivariate analysis of variance. We estimated incidence rate ratios (IRRs), incidence rate differences, and corresponding 95% CIs for each cause of death separately using Poisson regression models with robust standard errors. All analyses were conducted at the cluster level (the level of randomization). As a secondary analysis, we assessed the effect of azithromycin on all infectious cases of mortality using similar methods. All analyses were conducted in Stata v. 17.0 (StataCorp, College Station, TX). All statistical tests were two sided, with a *P*-value of <0.05 considered statistically significant.

## RESULTS

**Figure 1.**
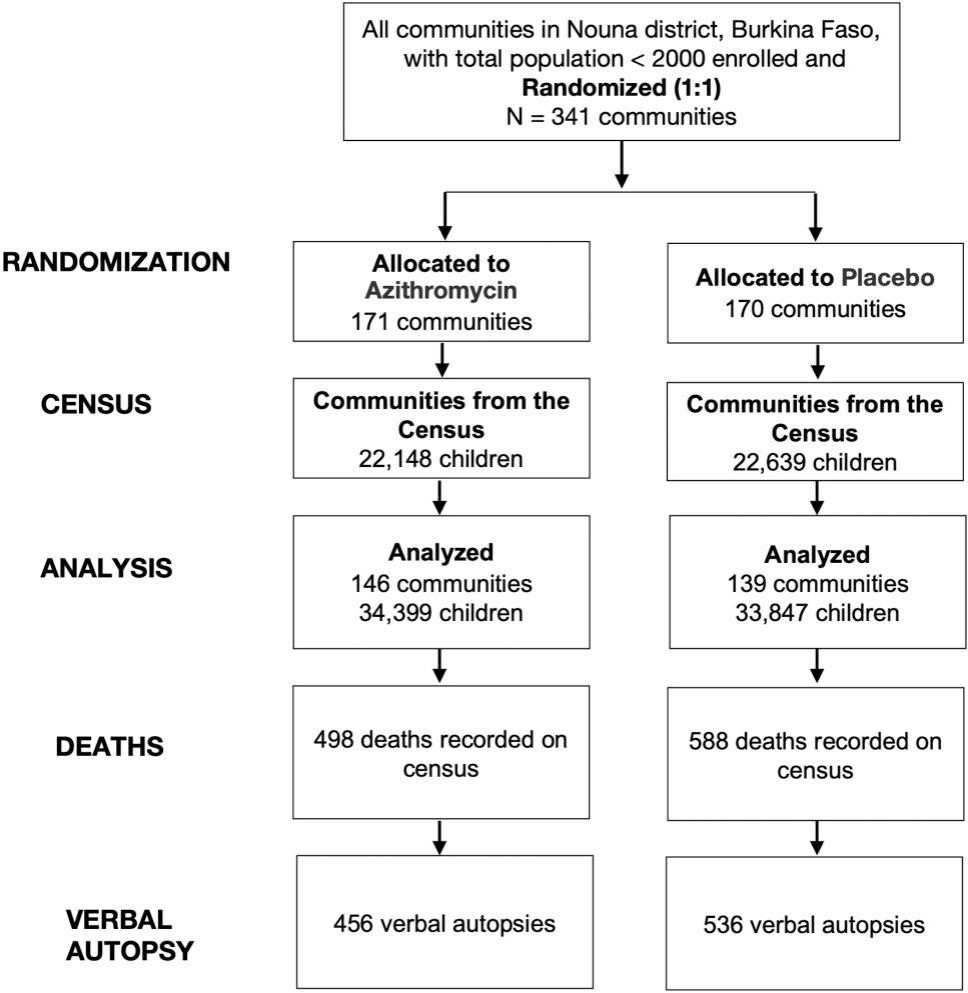
Consolidated standards of reporting trials diagram of study participation.

Of 341 communities originally randomized as part of the trial, 146 communities in the azithromycin group and 139 communities in the placebo group contributed person-time to the trial and were included in the analysis (Figure 1). Communities were treated from August 2019 through February 2023. Of the 341 originally randomized communities, 56 communities did not contribute person-time because of security concerns that arose during the conduct of the trial.^2^ During the baseline census, the distributions of age and sex were similar between children residing in communities in the azithromycin and placebo groups (Table 1). The primary outcome, all-cause mortality, has previously been reported.^2^ The primary outcome included 498 deaths in the azithromycin group and 588 deaths in the placebo group. Of these, verbal autopsies were available for 456 (91.6%) deaths in the azithromycin group and 536 (91.2%) deaths in the placebo group.

**Table 1:** Community characteristics at the baseline census

Characteristics	Azithromycin Group	Placebo Group
Number of communities	144	140
Number of children	22,148	22,639
Age group, months		
1–11	3,606 (16.3%)	3,580 (15.8%)
12–23	4,575 (20.7%)	4,709 (20.8%)
24–59	13,967 (63.1%)	14,350 (63.4%)
Sex		
Male	11,265 (50.9%)	11,390 (50.3%)
Female	10,883 (49.1%)	11,249 (49.7%)

**Figure 2.**
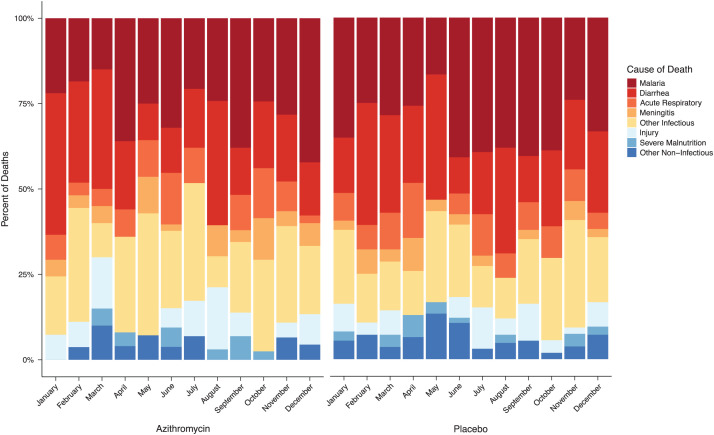
Cause of death as assigned by verbal autopsy in children living in communities randomized to azithromycin (left panel) and placebo (right panel) distribution by month of death.

**Figure 3.**
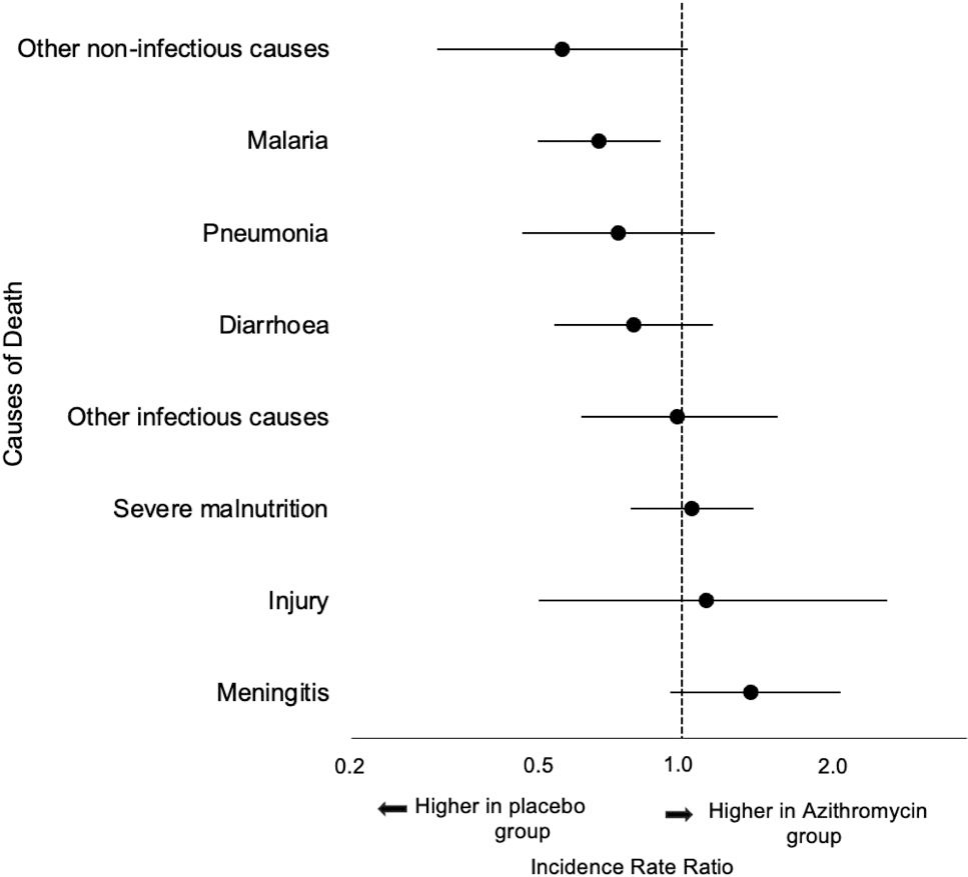
Effect of azithromycin compared with placebo on cause-specific mortality. Point estimates are incidence rate ratios, and bars are 95% CIs. Malaria was the only cause of death that was statistically significantly lower in the azithromycin group compared with the placebo group.

The overall distribution of deaths did not differ between azithromycin and placebo communities (*P* = 0.21). Infectious causes were the most common for all calendar months of the year (*N* = 866, 80%; over entire study period), with an increase in the number of malaria deaths in the high-malaria-transmission season (Figure 2). The most common cause of death per verbal autopsy was malaria (Table 2). There were 127 malaria-associated deaths in the azithromycin group (incidence rate of 2.3 malaria deaths per 1,000 person-years) and 181 malaria deaths in the placebo group (incidence rate of 3.4 malaria deaths per 1,000 person-years), corresponding to a 33% reduction in malaria mortality in azithromycin-treated communities compared with placebo (IRR, 0.67; 95% CI, 0.50–0.90) (Figure 3). Diarrhea was the second-most common cause of mortality, with 98 deaths (1.8 diarrhea deaths per 1,000 person-years) in the azithromycin group and 118 deaths (2.2 diarrhea deaths per 1,000 person-years) in the placebo group (IRR, 0.79; 95% CI, 0.54–1.16), followed by pneumonia and meningitis (Table 2). An analysis pooling all infectious causes of mortality into a single group (all-cause infectious mortality) found an infectious mortality rate of 7.1 deaths per 1,000 person-years in the azithromycin group and 8.8 deaths per 1,000 person-years in the placebo group, consistent with an approximately 20% reduction in infectious mortality in communities receiving azithromycin (IRR, 0.80; 95% CI, 0.64–1.01). The most common noninfectious cause of mortality was injury, and there was no evidence of a difference in injury-related mortality between groups (Figure 3; Table 2).

**Table 2:** Primary cause of death as assigned by verbal autopsy in communities randomized to azithromycin and placebo

Primary Causes of Death	Azithromycin		Placebo		Incidence Rate Ratio (95% CI)	*P*-Value	Incidence Rate Difference* (95% CI)	*P*-Value
	No. of Children (%)	Incidence per 1,000 Person-Years (95% CI)	No. of Children (%)	Incidence per 1,000 Person-Years (95% CI)				
Infectious causes								
Malaria	127 (25.5%)	2.27 (1.8–2.74)	181 (31.2%)	3.40 (2.67–4.12)	0.67 (0.50–0.90)	0.008	−1.13 (−2 to −0.26)	0.01
Diarrhea	98 (19.7%)	1.75 (1.26–2.25)	118 (20.3%)	2.22 (1.63–2.8)	0.79 (0.54–1.16)	0.23	−0.47 (−1.23 to 0.3)	0.23
Pneumonia	37 (7.4%)	0.66 (0.45–0.88)	48 (8.3%)	0.9 (0.6–1.21)	0.73 (0.46–1.17)	0.19	−0.24 (−0.61 to 0.13)	0.21
Meningitis	25 (5.0%)	0.45 (0.25–0.64)	17 (2.9%)	0.32 (0.17–0.47)	1.4 (0.73–2.67)	0.31	0.13 (−0.12 to 0.37)	0.31
Other infectious causes^†^	109 (21.9%)	1.95 (1.54–2.36)	106 (18.3%)	1.99 (1.52–2.46)	0.98 (0.71–1.34)	0.89	−0.04 (−0.67 to 0.58)	0.89
Noninfectious causes								
Injury	32 (6.1%)	0.57 (0.36–0.79)	27 (4.9%)	0.51 (0.33–0.68)	1.13 (0.67–1.89)	0.64	0.06 (−0.21 to 0.34)	0.65
Severe malnutrition	11 (2.2%)	0.2 (0.07–0.32)	10 (1.7%)	0.19 (0.07–0.31)	1.05 (0.42–2.63)	0.92	0.01 (−0.17 to 0.19)	0.92
Other noninfectious causes^‡^	17 (3.4%)	0.3 (0.16–0.45)	29 (5.0%)	0.54 (0.33–0.75)	0.56 (0.3–1.03)	0.06	−0.24 (−0.5 to 0.01)	0.06
Unspecified	42 (8.4%)	0.75 (0.51–0.99)	44 (7.6%)	0.83 (0.49–1.16)	0.91 (0.54–1.52)	0.71	−0.08 (−0.49 to 0.34)	0.72

* Difference in number of deaths per 1,000 person-years.

^†^ Other infectious causes include dengue fever (*n* = 22), HIV-related deaths (*n* = 7), nondengue hemorrhagic fever (*n* = 10), pertussis (*n* = 4), tuberculosis (*n* = 3), measles (*n* = 2), sepsis (*n* = 9), and tetanus (*n* = 1).

^‡^ Noninfectious causes include acute abdomen (*n* = 10), congenital and birth-related events (*n* = 9), epilepsy (*n* = 3), contact with venomous plant/animal (*n* = 14), liver failure (*n* = 2), sickle cell crisis (*n* = 1), and vascular events (*n* = 5).

## DISCUSSION

In this secondary analysis of verbal autopsy data from a cluster-randomized trial of biannual mass azithromycin distribution compared with placebo for the prevention of mortality in Burkina Faso, we found evidence of a reduction in malaria mortality as determined by verbal autopsy in children living in azithromycin communities compared with placebo communities. The primary analysis of the trial found an 18% reduction in all-cause child mortality in communities receiving azithromycin compared with placebo, a result consistent with the Niger site of the MORDOR trial.^1^^,^^2^ The CHAT trial was performed in the presence of SMC; communities in CHAT have received monthly SMC from July through October since 2014. In contrast, SMC distribution had not yet begun in the districts in Niger in which the original MORDOR trial was conducted. A previous household randomized, controlled trial comparing SMC distribution with and without azithromycin found no additional benefit of adding azithromycin to SMC treatment regimens.^16^ This led to a hypothesis that there may be no additional benefit of mass azithromycin when SMC is also being distributed given that malaria is a major cause of mortality in the study area, that SMC reduces malaria mortality, and that sulfadoxine may have nonmalarial activity against pathogens that contribute to child mortality.^5^^,^^17^ In the present analysis, malaria mortality was more common during the SMC season, and malaria mortality was significantly reduced in communities receiving azithromycin compared with placebo.

Azithromycin has weak antimalarial properties as it targets the plasmodial apicoplast.^7^^,^^8^ Previous studies have found limited evidence of an effect of mass azithromycin distribution for reduction in parasitemia and serologic responses to merozoite surface protein 1, a surrogate for malaria incidence.^9^^,^^10^^,^^18^^–^^23^ One study suggested a short-term reduction in malaria parasitemia after mass azithromycin distribution for trachoma,^21^ and other studies have documented small reductions in parasitemia in communities receiving azithromycin compared with placebo for the prevention of child mortality and in communities receiving biannual azithromycin compared with annual azithromycin for trachoma.^9^^,^^23^ However, other studies have found no effect of azithromycin on parasitemia,^22^ and individually randomized studies of single-dose azithromycin have not shown an effect of azithromycin on short- or longer-term parasitemia prevalence.^11^^,^^12^ Analysis of verbal autopsy data showed a 22% reduction in malaria mortality in the MORDOR trial,^3^ consistent with findings from the present study. An analysis of the Child Health and Mortality Prevention Surveillance (CHAMPS) study documented a high prevalence of bacterial coinfection in children who died of malaria.^24^ If direct effects of azithromycin on malaria are insufficient to reduce malaria mortality, it is possible that azithromycin distribution could reduce bacterial coinfections that may increase malaria-related mortality.

The use of verbal autopsy for assigning cause of death is prone to misclassification, which may bias results.^25^ In this study, malaria was the most commonly assigned cause of death per the verbal autopsy. Pneumonia was less common than expected based on cause-specific health care visits for children younger than 5 years old, for whom pneumonia is the most common,^26^^,^^27^ and cause of death estimates from sub-Saharan Africa.^28^ Deaths in the study area were identified by biannual census, often occurring outside of the health system with no physician-assigned cause of death information or diagnostic information available. Verbal autopsy has generally been shown to have low sensitivity but relatively higher specificity for assigning malaria mortality.^24^^,^^25^^,^^29^^,^^30^ A previous analysis of CHAMPS data showed 29% sensitivity and 87% specificity of the inSilicoVA algorithm for assigning malaria as a cause of death versus minimally invasive tissue sampling.^24^ Although we did not have a gold-standard method of assigning cause of death in the present study, if the CHAMPS results are generalizable to the CHAT study, this would suggest that malaria mortality would be undercalled rather than overcalled. Because the study assignment in the current trial was masked and because the study was placebo controlled, we anticipate that misclassification of cause of death owing to the use of verbal autopsy would be nondifferential. If misclassification was nondifferential with respect to treatment assignment, we anticipate that this would bias results toward the null and that the true effects would be greater than those observed in this study.

In addition to limitations because of potential misclassification owing to the use of verbal autopsy and the absence of a gold-standard method of assigning cause of death, there are several limitations to consider in this analysis. First, even in high-mortality settings, mortality is a rare outcome. CIs were wide for all causes of mortality, especially those that were less common. Although the finding is consistent with the MORDOR study, it is possible that the reduction in malaria mortality was because of chance. For example, although the CI included one, there appeared to be a reduction in mortality because of noninfectious causes that were not owing to accident (e.g., snake bite) in azithromycin-treated communities. However, this is most likely a chance finding because of the lack of biological plausibility for an association between azithromycin distribution and noninfectious causes of mortality. Nonmalarial infectious causes of mortality, including pneumonia and diarrhea, were nonstatistically significantly lower in azithromycin communities, but CIs were wide because of low statistical power. An analysis considering all infectious causes of mortality found approximately 20% reduced risk of infectious mortality in communities receiving azithromycin compared with placebo, although the CI narrowly included the null. Additional stratified analyses (for example, by SMC season) may provide additional insights into the impact of azithromycin on cause-specific mortality, but the study was not powered to support such analyses. Second, verbal autopsies were not available for all deaths. Verbal autopsies are performed several months after the death occurred to honor the mourning period. Families may be untraceable after this period. The percentage of deaths for which a verbal autopsy was available was similar between the two study arms, and missingness was not differential by arm. Third, some clusters in the district were excluded from the study owing to insecurity. This could potentially introduce selection bias if the exclusion was differential with respect to both the randomized treatment assignment and the outcome. Because of the masked, randomized nature of the trial, we do not anticipate that this introduced substantial selection bias, but as these communities did not participate in the baseline census, we are unable to assess the distribution of baseline characteristics by cluster. Fourth, results may not be generalizable to settings with a different distribution of causes of mortality. Although results of the effect of biannual azithromycin distribution on mortality have been broadly consistent in studies conducted in the Sahel (e.g., the Niger site of MORDOR and the CHAT study), these results may not be generalizable outside of the Sahel.^31^^,^^32^

## CONCLUSION

In this analysis of cause-specific mortality, we found a reduction in verbal autopsy-assigned malaria mortality in communities receiving biannual mass azithromycin distribution to children ages 1–59 months old compared with placebo. This finding was consistent with previous studies of mass azithromycin distribution for the prevention of child mortality in the Sahel. This study suggests that distributing azithromycin twice a year could offer additional benefits in reducing mortality, even in areas where SMC is being distributed. Further studies with gold-standard malaria and malaria mortality assessments are warranted.
